# Influences of Migrant Construction Workers’ Environmental Risk Perception on Their Physical and Mental Health: Evidence from China

**DOI:** 10.3390/ijerph17207424

**Published:** 2020-10-12

**Authors:** Yao Jiang, Huawei Luo, Fan Yang

**Affiliations:** 1 Department of Accounting, School of Management, Sichuan Agricultural University, Chengdu 611130, China; s20175706@stu.sicau.edu.cn; 2 Department of Labor and Social Security, School of Public Administration, Sichuan University, Chengdu 610065, China

**Keywords:** migrant construction worker, risk perception, physical health, mental health, environmental welfare

## Abstract

Employing Chinese General Social Survey 2013 data (*N* = 678), this study examines the influences of migrant construction workers’ environmental risk perception (ERP) on their physical and mental health. The ERP of migrant construction workers is characterized by six dimensions: perceptions of air pollution, industrial waste pollution and noise pollution at working sites, and perceptions of domestic waste pollution, water pollution and food pollution at living sites. The results indicate that migrant construction workers with stronger ERP have better physical and mental health. The results also suggest the influences of ERP on the physical and mental health of migrant construction workers with different gender and age (<50 and ≥50 years) are heterogeneous. Perceptions of industrial waste pollution, noise pollution and domestic waste pollution significantly affect female workers’ physical health, but not that of male workers. The six dimensions of ERP all significantly influence male workers’ mental health, while except for domestic waste pollution perception, the other perceptions do not influence that of female workers. Perceptions of air pollution, domestic waste pollution, and water pollution significantly influence physical health of workers aged 50 and above, while those of ERP do not work on that of workers younger than 50. Perception of food pollution significantly influences mental health of workers younger than 50, but not that of workers aged 50 and above. The seemingly unrelated regression shows the results in this paper are robust.

## 1. Introduction

Risk exists universally across the world and may cause undesirable consequences. Risk perception has been described as people’s subjective and experiential judgements on the magnitudes or likelihoods of negative outcomes resulting from currently faced or anticipated risks [1,2,3]. The behaviors of individuals are mainly determined by self-cognition [4]. Compared with individuals with a lower level of risk perception, those with a higher level of risk perception are more willing to adopt self-protective intervention measures to defend against or avoid possible undesirable consequences [5]. Environmental pollution may affect individual health and is the main aspect of environmental risk. Examples are air pollution, industrial waste pollution, noise pollution, domestic waste pollution, water pollution, and food pollution [6,7]. Based on people’s intuitions and experiences, environmental risk perception (ERP) can trigger either rejection or adoption of self-precautionary or self-protective health behaviors by judging the severity of environmental risk [8,9,10,11]. Therefore, ERP may significantly influence individual health.

This study focuses on the influences of ERP on the physical and mental health of a vulnerable group: migrant construction workers in China. In this paper, ERP refers to migrant construction workers’ subjective perception toward the severity of environmental pollution problems at both their working and living sites. Its function as a trigger factor for either the adoption or rejection of self-precautionary health behaviors is investigated when workers are faced with problems associated with environmental pollution [12,13].

Migrant construction workers appeared during the process of Chinese urbanization and industrialization. In this process, a large labor force surplus migrated from rural areas, where their *hukou* (household registration) was registered with the government, to urban places for non-agricultural work. The construction industry has the dominant characteristics of being highly labor-intensive and requiring learning by doing, while it does not impose high requirements of education and professional skills for job candidates [14]. In this setting, the construction industry has become a common destination industry for migrant labor workers with low non-agricultural technical attainment [15]. The general traits of migrant construction workers include their mainly low educational level and their engagement in heavy labor and short-term work [16,17]. Migrant labor workers from rural areas are the major labor suppliers of the construction industry in cities. At the end of 2018, there were 55.369 million construction employees in China, most of which, about 53.639 million, were migrant construction workers from rural areas [18].

The emergence of migrant construction workers has promoted a redistribution of labor resources and has greatly improved labor productivity [19]. These workers are significant contributors to prolonging the miracle of China’s economic growth [20,21]. However, to a large extent, migrant construction workers bear the negative externality of environmental pollution brought by economic growth [22]. They are constantly exposed to dirty, demanding and dangerous working and living environments. In addition to risky construction activities, environmental risks leave them firmly in a vulnerable position [23,24]. Specifically, air pollution, industrial waste pollution, and noise pollution affect their working sites and domestic waste pollution, water pollution, and food pollution affect their living sites [25,26,27].

With regard to air pollution, migrant construction workers breath air containing excessive amounts of hazardous dust materials, such as silica [28]. The hazards of dust on physical health are mainly reflected in respiratory diseases, such as pneumoconiosis, chronic obstructive pulmonary disease and asthma [29,30,31]. In China, a total of 19,524 cases of occupational respiratory diseases were reported by the end of 2018, including 19,468 cases of occupational pneumoconiosis, mainly distributed in the mining industry and the construction industry [32].

In addition to air pollution, industrial waste pollutants (including e-waste) also affect construction sites [33]. Studies have reported that in regions where industrial waste pollution is serious, the livers and lungs of migrant construction workers are damaged to varying degrees [34]. Furthermore, female construction workers’ breast milk, serum, and cord blood show higher levels of industrial waste pollutants than workers in other areas [35]. This indicates that industrial waste pollution poses an enormous health threat to both migrant construction workers and their following generations.

Construction noise is the most persistent physical contaminant at working sites [36]. The maximum emission standard of environmental noise at the boundary of construction sites in China is 70 dB during the day, and 55 dB at night [37]. However, migrant construction workers are often exposed to machines, which frequently release noise exceeding the stipulated maximum emission standard during working time [38]. Noise pollution can have a series of detrimental effects on workers, the most common effect being irreversible loss of hearing [39,40]. Other undesirable health outcomes include mental problems resulting from sleep disturbance [41]. In China, hearing protective equipment is rarely provided to migrant construction workers [28].

At their living sites, migrant construction workers face severe levels of domestic waste pollution around the accommodations. Restrained by living cost in urban areas, most migrant construction workers live in temporary housing made of iron plates and foam or in rental housing that had been abandoned by local residents, which are small, humble, and are almost only used for cooking and sleeping [25,42]. At the end of 2019, the average per capita living space of migrant workers in a Chinses city with a population of five million was only 16.5 m^2^ [43]. Migrant construction workers also have a low educational level and 70% only have junior high school education or below [18]. Most of these workers lack the environmental literacy required for refuse classification [33,44]. Moreover, their residences vary with the places of construction project implementation, and they are flowing and short-term residents of their living areas. Therefore, most of these migrant construction workers litter domestic waste at will and do not pay attention or consider the long-term need to protect their living environments [45].

Water is the source of life and an indispensable substance in human life. Pollutant sources (i.e., hazardous dust, industrial waste, noise, and domestic waste pollutants) on land or in the air can naturally release into micro-environments by precipitation runoff, which might eventually result in surface or ground water pollution [46,47]. Cholera, diarrhea, dysentery, and hepatitis A are directly linked to unhygienic and contaminated drinking and living water [48]. It has been reported that migrant construction workers in China routinely use well water for cooking, bathing, and cleaning rather than purified tap water [49]. Using unpurified well water (with its high likelihood of containing pollutants) for drinking and living can lead to contracting diseases [50].

Additionally, the diet of migrant construction workers is unhealthy to some extent. Migrant construction workers are different from general nine-to-five workers. They are engaged in short-term work, are paid daily, and have to complete the construction work within a prescribed time [51]. To save time, most employers provide working meals to migrant construction workers. However, because of cost considerations, employers generally do not take pollution-free, healthy, and green choices into account [52]. Among numerous food poisoning cases in China, the food pollution suffered by migrant construction workers mainly includes heavy metals in flour, seafood, and algal products, food borne pathogenic bacteria in raw or cooked meat and seafood, and pesticide residue pollution in vegetables [53].

To remind policy makers and employers the environmental risks migrant construction workers are exposed to, many published studies have objectively and quantitatively assessed the magnitude of environmental risks. These environmental risks include air pollution, industrial e-waste pollution, and occupational noise pollution at their working sites [20,25,28,54,55]. For example, a study taking currency as unit, quantitatively assessed workers’ health damage caused by construction dust, by employing a life cycle environmental impact assessment model and a disability adjusted life year model, and the results revealed the health damage caused by per unit earthwork construction approximate to $169 thousand every year [55]. Among these quantitative studies, it a consensus has been reached that the severity and hazards of the environmental risks migrant construction workers are exposed to need to receive more attention [56,57].

Although, objective and quantitative assessments on environmental risks are clearly part of improving workers’ health, migrant construction workers are the direct perceiver of environmental risks. Guided and dominated by their ERP, they either adopt or reject self-precaution behaviors against undesirable health outcomes that result from the environmental risks [4]. A high ERP is more likely to let migrant construction workers spontaneously know hazards of a certain environmental pollution problem, and adopt self-precautionary health behavior as their capacity allows. Examples are wearing masks and hearing protective equipment, which may improve their health more efficiently than passively waiting for the government and their employers to take relevant measures. In contrast, migrant construction workers with low ERP reckon that environmental risks are not sufficiently severe to pose a threat to their health. They may do not take self-precautionary measures and even refuse to implement protection measures provided by the employers or government for them. For instance, despite the mandatory requirement to wear helmets at construction sites, workers are still disabled or die every year because they do not wear safety helmets [24]. As a result, protection measures are weakened because of their low ERP, so as to their health status declines.

Therefore, the main aims of this paper are to understand the situation of migrant construction workers’ ERP at their construction working sites, including air pollution perception, industrial waste pollution perception, and noise pollution perception. Furthermore, this paper also focuses on the ERP of migrant construction workers at their living sites, including domestic waste pollution perception, water pollution perception, and food pollution perception. Moreover, this study probes the relationship between the ERP of migrant construction workers and their health to provide a risk perception path for the health improvement of migrant construction workers. This is not only conducive to helping migrant construction workers to obtain equal environmental benefits, but it is also beneficial for urban labor supply and urban development.

This paper fills a number of the research gaps in current studies on ERP of migrant construction workers. First, in addition to focusing on migrant construction workers’ ERP at their working sites as studied previously [35,58], this paper focuses on migrant construction workers’ ERP at their living sites. Second, since health is a multidimensional concept, this paper observes the influences of migrant construction workers’ ERP on both their physical and mental health. Finally, gender and age have been shown to cause risk perception bias [59]. As such, this paper particularly focuses on the heterogeneous influences of ERP on the physical and mental health of migrant construction workers in different gender and age groups.

## 2. Methods

### 2.1. Survey Design

The dataset employed in this paper was extracted from the Chinese General Social Survey 2013 (CGSS2013). The CGSS was initiated by Renmin University of China and Hong Kong University of Science and Technology. It is the first national representative survey dataset, implemented by academic institutions in China. The CGSS can also be regarded as the Chinese counterpart of the General Social Survey of the USA [60]. The CGSS aims to collect data in various areas of Chinese people’s socio-demographic characteristics, political and environmental perceptions, as well as attitudes and behaviors. It is a public dataset all researchers can use by applying to the National Survey Research Center.

The CGSS2013 employed a stratified multi-stage probability proportional to size (PPS) sampling design. The sampling structure of the CGSS2013 was based on the 2012 national population data. The samples of the CGSS were drawn from households from 31 provincial units in mainland China. There are three levels in the CGSS2013 PPS sampling frame: county is the primary sampling unit, community is the secondary sampling unit, and household is the tertiary sampling unit. In a randomly selected county, four community-level units were randomly selected. In a selected community-level unit, 25 households were sampled with the PPS method. Consequently, 11438 valid respondents from 400 community-level units in 140 counties were selected for the CGSS2013.

As a non-official survey project, the CGSS requires voluntary participation. Respondents are informed of the objectives and purposes of the survey projects before they begin to be interviewed. In addition, the survey team signs written promises with the respondents to keep personally identifiable information confidential. Respondents involved in the CGSS2013 are with various individual characteristics (such as age, marriage status, industry, income, educational level, and ethnicity) and family characteristics (such as family income, family size, and family expenditure) [61].

### 2.2. Sample Selection

According to the needs of this study, the original samples are screened. The conceptional definition of a migrant worker given by the National Bureau of Statistics (NBS) of China is that, migrant workers originate from rural areas where their *hukou* is registered, and who are engaged in non-agricultural work in urban areas for six months and longer and have an educational level of junior college or below [18,43]. As such, first, the full sample was divided into three groups based on their *hukou*, i.e., rural migrants, urban migrants and urban locals. Rural migrants have rural *hukou* and reside in cities, urban migrants have urban *hukou* but do not reside in the cities where their *hukou* is registered, and urban locals have local urban *hukou*. According to the conceptional definition of a migrant worker given by the NBS, the data for urban migrants and urban locals were excluded, and samples were restricted to rural migrants who engaged in non-agricultural work in urban areas for six months and longer, with an educational level of junior college or below. Second, based on the industrial classification for national economic activities in China (GB/T 4754—2017) [62], the samples were limited to respondents who worked in the construction industry. Finally, to decrease estimation bias as much as possible, invalid samples were removed. Specifically, samples with the following characteristics were excluded: key questions without answer, answer of “inapplicable, unclear”, or apparent logical contradictions. The final samples contained 678 samples of migrant construction workers.

### 2.3. Measurements

#### 2.3.1. Dependent Variable

Two dimensions (physical health and mental health of migrant construction workers) were measured as dependent variables. To assess physical health, survey participants were asked, “How would you rate your health status (variable named physical health)?” The answer was measured by a five-point Likert scale ranging from “1” to “5”. The choice of “very unhealthy” was coded as 1, “somewhat unhealthy” as “2”, “normal” as “3”, “somewhat healthy” as “4”, and “very healthy” as “5”.

With regard to mental health, the frequency of experiencing depression and hopelessness was used to reflect mental health. For instance, a study with the goal to identify the relationship between public mental health and the prevalence of COVID-19, employed the number of days that respondents experienced depression and hopelessness in the previous two weeks to measure mental health [63]. Furthermore, a study to test for depression and associated factors of migrant workers in China has regarded the frequency of depression as a relevant reference for clinical symptoms of depression [64]. As such, in this paper, survey respondents were asked, “How often did you experience depression and hopelessness in the previous two weeks (variable named mental health)”? The answer was similarly measured by a five-point Likert scale ranging from “1” to “5”. The choice of “always” was coded as “1”, “often” as “2”, “sometimes” as “3”, “seldom” as “4”, and “almost never” as “5”.

#### 2.3.2. Explanatory Variable

To measure ERP, many previous studies have employed Likert scales [65,66,67]. A study that probed the correlation between public ERP and well-being has measured the ERP by asking respondents, “How would you rate the severity of environmental pollution?” Answers were made on an 11-point scale from 0 (not severe) to 10 (extremely severe) [68].

In this paper, to measure the six dimensions of ERP, including perceptions of air pollution, industrial waste pollution, noise pollution, domestic waste pollution, water pollution, and food pollution, the survey participants were asked, “How would you rate the severity of air/industrial waste/noise/domestic waste/water/food pollution in the environment you are working or living?” The answers were measured by a six-point Likert scale ranging from “0” to “5”. The answer of “no pollution at all” was coded as “0”, “not severe at all” as “1”, “not so severe” as “2”, “normal” as “3”, “somewhat severe” as “4”, and “extremely severe” as “5”.

#### 2.3.3. Control Variables

In addition to environmental pollution, studies have also documented that migrant construction workers’ health is affected by their socio-demographic characteristics, such as gender, age, ethnicity, education, income, working year, environmental literacy, and frequency of mass media usage [25,69,70,71,72,73,74,75,76,77,78]. Therefore, the influences of these variables on migrant construction workers’ health were controlled.

Specifically, previous studies have shown that females have better physical health status than males, but worse mental health than males [69,70]. In this paper, gender was a dummy variable with “1” representing male, and “0” representing female. In general, individual health declines with the increment of age and working years [71]. Age and working year were both continuous variables. Migrant construction workers of different ethnicities have different socio-cultural contexts. A number of minorities in China hold a fatalistic attitude to disease, which may cause their diseases to further deteriorate [72]. In this paper, ethnicity was a dummy variable with “1” representing the Han ethnicity, and “0” representing any non-Han ethnicity. Educational level is closely linked to self-care behavior and health care accessibility [73,74]. In this paper, education was a continuous variable and measured by the years of schooling. As a classical factor to affect health, income was also tested. It has been shown that workers with low income tend to save money to support family, rather than spending money on health services for themselves [25]. Therefore, migrant construction workers with a lower income level may have a weaker sense of health, and worse health status compared with workers with higher income. The variable of income was a continuous variable, and was measured via the total wage income of respondents in 2012. In the later data analysis, the logarithm of income was used. Environmental literacy is the capacity to perceive and interpret the environment, which can trigger people to take appropriate action to maintain, restore, or improve their health [75,76]. Environmental literacy was a continuous variable in this study, and was measured by the scores of the respondents’ answers to 10 questions related to environmental and health knowledge (see Appendix A). One correct answer scored one point, whereas incorrect answers or answers of “not clear” scored zero points. Furthermore, many researches on migrant construction workers’ health have assessed the influence of mass media [77,78]. It has been widely recognized that mass media can improve migrant construction workers’ health. The frequency of mass media usage was measured by respondents’ usage frequency of three types of mass media carriers: television, internet, and phone. The survey participants were asked, “How often do you use television / internet / phone in your daily life?” The answers were measured by a five-point Likert scale ranging from “1” (almost never) to “5” (always).

In addition, the impact of regional fixed effects on the health of migrant construction workers has also been controlled. Region was a dummy variable, measured by the provinces in which respondents worked.

### 2.4. Data Analysis Strategy

The two measured outcomes of (1) physical health and (2) mental health of migrant construction workers were employed as dependent variables. The six dimensions of ERP (i.e., (1) air pollution perception, (2) industrial waste pollution perception, (3) noise pollution perception, (4) domestic waste pollution perception, (5) water pollution perception, and (6) food pollution perception) were used as explanatory variables. Because the two dimensions of dependent variable were ordered discrete data, the ordered probit (Oprobit) regression models were applied to estimate the influences of ERP on migrant construction workers’ physical and mental health.

In general, an Oprobit model is expressed as:

(1) $$y_{i}^{*}=x_{i}^{\prime }\beta +\varepsilon ,$$

Assuming the selection rule of $y_{i}$:

(2) $$y_{i}=\{\begin{array}{cc} 1 & y_{i}^{*}<\mu_{1}\\ 2 & \mu_{1}\leq y_{i}^{*}<\mu_{2}\\ \ldots & \ldots \\ J & \mu_{J-1}\leq y_{i}^{*} \end{array},$$

where, $y_{i}^{*}$ cannot be observed directly and $y_{i}$ represents the observed counterpart of $y_{i}^{*}$. $\mu_{1}<\mu_{2}<\ldots <\mu_{J-1}$ are cut points. $x_{i}^{\prime }$ represents the dependent variable vector. β represents the coefficient vector. β and $\mu_{j}$ (j=1,2,3…J) are estimated by maximum likelihood estimation.

In this paper, the specific Oprobit models can be written as follows:

(3) $$\mathrm{Physical}\;\mathrm{helat}h_{i}=\alpha_{1}+\beta_{1}ERP_{i}+\gamma X+\varphi_{1},$$

(4) $$\mathrm{Mental}\;\mathrm{healt}h_{i}=\alpha_{2}+\beta_{2}ERP_{i}+\gamma X+\varphi_{2},$$

where, $\mathrm{Physical}\;\mathrm{healt}h_{i}$ and $\mathrm{Mental}\;\mathrm{healt}h_{i}$ (i.e., the dependent variables) represent the physical and mental health status of the i−th migrant construction worker. $ERP_{i}$ includes the independent variables and consists of perceptions of air pollution, industrial waste pollution, noise pollution, domestic waste pollution, water pollution, and food pollution. $\alpha_{1}$ and $\alpha_{2}$ represent the intercept terms. $\beta_{1}$ and $\beta_{2}$ represent the influences of ERP on the physical and mental health of migrant construction workers, respectively. If $\beta_{1}$ and $\beta_{2}$ are statistically significant and positive, migrant construction workers with stronger ERP have better physical and mental health. X represents the set of control variables. $\varphi_{1}$ and $\varphi_{2}$ are random perturbed terms.

To ensure the robustness of the estimation results, a robustness check was conducted by using the seemingly unrelated regression (SUR) estimation. In general, individual physical and mental health were closely related [75]. Both may be determined by unobserved variables. Therefore, the covariance of the perturbed terms between the physical and mental health equations may be non-zero. SUR estimation is the commonly applied method to effectively recheck the results of two Oprobit regression models in which the dependent variables are related [79]. As such, SUR was employed to joint estimated model (3) and (4), to recheck the influences of six dimensions of ERP on migrant construction workers’ physical and mental health. The analysis was implemented by the statistical software Stata version 15 (Stata Crop. LP., College Station, TX, USA).

## 3. Results

### 3.1. Description Analysis

The distribution of variables (*N* = 678) is provided in Table 1. With regard to the two dependent variables, the mean value of the physical health of migrant construction workers was 4.10 (median 4, IQR 4 to 5, SD = 0.87), and the mean value of the mental health of migrant construction workers was 4.11 (median 4, IQR 4 to 5, SD = 0.88).

With regard to the ERP that may influence migrant construction workers’ physical and mental health, the mean values of air pollution perception, industrial waste pollution perception, and noise pollution perception were 2.81 (median 3, IQR 2 to 4, SD = 1.55), 2.55 (median 3, IQR 1 to 4, SD = 1.57), and 2.78 (median 3, IQR 2 to 4, SD = 1.50), respectively. The domestic waste pollution perception, water pollution perception and food pollution perception had average values of 2.74 (median 3, IQR 2 to 4, SD = 1.44), 2.86 (median 3, IQR 2 to 4, SD = 1.50), and 2.81 (median 3, IQR 2 to 4, SD = 1.47), respectively.

With regard to control variables, among all subjects, 15.19% were female, while 84.81% were male. The ages of respondents ranged from 17 to 65 years, with an average value of 41.02 years. The ethnicity showed that 92.63% of respondents were of Han ethnicity, while 7.37% were of non-Han ethnicity. The average years of education of respondents were 9.27 years, with a minimum of 0 (illiterate) and a maximum of 15 (junior college) years. The average logarithm of income of migrant construction workers in 2012 was 10.21, with a minimum of 7.6 and a maximum of 12.9. The working year of respondents ranged from 1 to 50 years, with an average value of 15.24 years. The mean score of environmental literacy was 4.82, which showed that, on average, respondents answered 4.8 questions (the total number of questions: 10) on environment and health correctly. Regarding the frequency of mass media usage, the average values of using television, internet, and phone were 4.13, 2.68, and 1.93, respectively.

### 3.2. Influences of ERP on Migrant Construction Workers’ Physical and Mental Health

The results of the influences of six dimensions of ERP on migrant construction workers’ physical and mental health are listed in Table 2. Technical diagnostic tests were conducted, and the Oprobit regression models were found to fit well.

Table 2 shows that perceptions of air pollution, industrial waste pollution, noise pollution, domestic waste pollution, water pollution, and food pollution were all significantly and positively associated with migrant construction workers’ physical health. It indicates that the higher the perceptions of air pollution, industrial waste pollution, noise pollution, domestic waste pollution, water pollution, and food pollution of workers, the better their physical health.

With regard to the influences of the ERP on migrant construction workers’ mental health, Table 2 shows that perceptions of air pollution, industrial waste pollution, noise pollution, domestic waste pollution, water pollution, and food pollution were also significantly and positively associated with the mental health of migrant construction workers. This indicates that the higher the perceptions of air pollution, industrial waste pollution, noise pollution, domestic waste pollution, water pollution, and food pollution in migrant construction workers, the more likely they were to have a better mental health.

With regard to the results of control variables, in terms of their influences on workers’ physical health, male migrant construction workers had worse physical health status than female. Older migrant construction workers had worse physical health than younger workers. Income and using frequency of the television significantly and positively affected migrant construction workers’ physical health. With regard to the influences of control variables on workers’ mental health, age and using frequency of the internet were significantly and positively associated with workers’ mental health.

### 3.3. Influences of ERP on Migrant Construction Workers’ Physical and Mental Health for Different Gender Groups

Gender induces perception bias and differences of health [11]. Therefore, the samples were grouped into two sub-groups (females and males) to assess the heterogeneous influence of six dimensions of ERP on the physical and mental health of different gender groups. Oprobit regression models were employed, and the establishment and estimation procedures of the models for different gender groups were the same as the Oprobit model for full samples. Technical diagnostic tests were conducted. The models were found to have a good fit and the results are reported in Table 3.

Table 3 shows that the six dimensions of ERP exerted heterogeneous influences on the physical health of different gender groups. Perceptions of industrial waste pollution, noise pollution, and domestic waste pollution were significantly and positively correlated with female workers’ physical health, while it was not correlated with that of male workers. However, perceptions of water pollution and food pollution were significantly and positively associated with male workers’ physical health, but not with that of female workers.

With regard to the heterogeneous influences of ERP on the mental health of different gender groups, the results provided in Table 3 show that the six dimensions of ERP all exerted significant and positive influences on male workers’ mental health. However, only domestic waste pollution perception had a significant and positive influence on the mental health of female workers, while the other five dimensions of ERP did not significantly correlate with their mental health.

Regarding the results of control variables in Table 3, for female workers, the working year and frequency of using the internet were significantly and positively associated with both their physical and mental health. For male workers, age was significantly and negatively correlated with both their physical and mental health, while income and usage frequency of television were significantly and positively related to their physical and mental health.

### 3.4. Influences of ERP on Migrant Construction Workers’ Physical and Mental Health for Different Age Groups

Age also may lead to perception bias [80]. Therefore, the samples were grouped into two sub-groups: respondents below 50, and those 50 and over. These groups were used to estimate the heterogeneous influences of ERP on the physical and mental health of these two age sub-groups. Oprobit regression models were employed, and the establishment and estimation procedures of the models for different age groups were the same as those employed for the Oprobit model for the full samples. The results are listed in Table 4.

With regard to workers’ physical health, perceptions of air pollution, domestic waste pollution, and water pollution exerted significant and positive influences on physical health of workers aged 50 and above, while the three types of ERP were not associated with the physical health of the age group below 50. However, food pollution perception was significantly and positively associated with the physical health of the age group below 50, while it had no significant influence on the physical health of workers aged 50 and above.

With regard to the mental health of different age groups, food pollution perception had a significant and positive influence on the age group below 50, but were not correlated with the mental health of the age group of 50 and above. Furthermore, for the control variables, income and television were significantly and positively linked to both physical and mental health of migrant construction workers aged 50 and above.

### 3.5. Robustness Check

As mentioned above, SUR was conducted to check the robustness of the estimation results. The results estimated by SUR are provided in Table 5. As shown in Table 5, the results of the Breusch-Pagan (BP) test at the 1% significance level refused the null hypothesis that the residuals of both models were independent from each other (Chi^2^ = 8.372, Pr = 0.000). This indicates that the estimation of SUR was effective. The influences of six dimensions of ERP on migrant construction workers’ physical and mental health were consistent with the results of Table 2. Specifically, perceptions of air pollution, industrial waste pollution, noise pollution, domestic waste pollution, water pollution, and food pollution were all significantly and positively associated with migrant construction workers’ physical and mental health. In conclusion, to some extent, the results in Table 2 were robust, and the six dimensions of ERP imposed influences on workers’ physical and mental health.

## 4. Discussion

By employing representative survey data from China, this paper examines the influences of six dimensions of ERP on migrant construction workers’ physical and mental health. These six dimensions include: air pollution perception, industrial waste pollution perception and noise pollution perception at working sites, as well as domestic waste pollution perception, water pollution perception and food pollution perception at living sites. Based on previous studies, migrant construction workers’ ERP was extended from the working sites to also include the living sites.

As expected, perceptions of air pollution, industrial waste pollution, and noise pollution at migrant construction workers’ working sites all significantly and positively influenced their physical and mental health. Also, migrant construction workers’ domestic waste pollution perception, water pollution perception, and food pollution perception at living sites significantly and positively affected their physical and mental health. As many studies have already substantiated, high nonfatal injuries or illness, and mortality of migrant construction workers were often related to their low occupational risk perception [24,45,57]. The results of this paper not only show that the ERP in their working sites can affect their health, but that the ERP in their living sites also affects their health. Therefore, in addition to improve the natural working and living environments, correctly guiding and reinforcing migrant construction workers’ ERP in working and living sites will be a potential way for both the government and non-governmental organizations to improve the physical and mental health of migrant construction workers.

Due to gender induced risk perception bias [81], heterogeneous influences of ERP on physical and mental health were found in various gender groups in this paper. With regard to physical health, water pollution and food pollution perceptions influence male migrant construction workers’ physical health significantly and positively, but not that of female workers. With regard to mental health, perceptions of air pollution, industrial waste pollution, noise pollution, domestic waste pollution, water pollution, and food pollution all significantly and positively influenced male workers’ mental health. However, except for domestic waste pollution perception, none of the other dimensions of ERP was significant for female workers’ mental health.

Previous studies found that women have more awareness and perception of health-related warning signs and use health services more frequently than men to maintain their health [82,83]. However, why the most ERP of female migrant construction workers do not significantly influence their physical or mental health in this paper? In general, a female worker is engaged in heavy and risky labor work at a construction site, which indicates that she may originate from a family in straitened circumstances, which requires the female to support the family [84]. Female construction workers shoulder heavier economic pressure than male workers [85,86]. Under this setting, female migrant construction workers have to ignore environmental risks they are exposed to, such as food and water pollution, and reject acting out of self-precaution and self-protection, as this might increase living costs. The family stress and constrains of urban living costs may lead to the failing of the influences of ERP on female workers’ physical or mental health. Female migrant construction workers have just as significantly contributed to the rapid development of China as male workers, but they are marginalized in the current social structure [87]. These results are a reminder that those female migrant workers at construction sites who shoulder the heavy burdens of their family as well as the heavy physical construction work should not be neglected.

The heterogeneous influences of ERP on physical and mental health in different age groups were also identified. With regard to physical health, perceptions of air pollution, domestic waste pollution, and water pollution exert significant and positive influences on the physical health of migrant construction workers aged 50 and above, while not influencing workers younger than 50. However, food pollution perception has a significant and positive influence on the physical health of migrant construction workers aged below 50, but not in workers aged 50 and above. With regard to mental health, food pollution perception significantly and positively affects the mental health of workers aged below 50, but not that of workers aged 50 and above.

Younger people often underestimate the environmental risks they are currently facing or might face, while older people tend to overestimate these [88]. With increasing age and richness of life experience, older people are more likely to contract, or witness other people contract diseases as a result of environmental risks [89]. Consequently, older migrant construction workers are inclined to take more self-precautionary and self-protection measures to maintain their health than younger workers. In contrast, younger migrant construction workers have less life experience, and underestimate environmental risks to a high extent. This leads to their perceptions of air pollution, domestic waste pollution, and water pollution to be unrelated with their physical health.

However, with the continued socio-economic advancement, the times of struggling to fill the stomach have passed, and thus, the younger workers care more about the quality of their food than older workers [90]. Therefore, the younger workers have more susceptive perception on food pollution than older workers.

Overall, compared with older workers, the influences of ERP on younger construction migrant workers’ health are weaker, which can be explained because younger construction migrant workers understate environmental risks. At the end of 2019, migrant workers below the age of 50 accounted for 75.1% of the total number of migrant workers in China [43]. These younger migrant construction workers should receive more environmental risk education to further improve their self-prevention awareness. Specific measures may include the handing out of leaflets about environmental risks, and the popularization of health- and environmental-related information through mass media carriers they use frequently, such as television, the internet, or WeChat.

With regard to control variables, migrant construction workers’ frequency of television and internet usage is particularly effective in improving their physical or mental health. Because of their popularity and entertainment value, television and internet can be used as a carrier of environmental risk and health-related information for migrant construction workers with low educational level. Environmental risk and health-related information can guide migrant construction workers’ healthy lifestyle, and improve their physical or mental health [91,92]. Therefore, in order to further improve migrant construction workers’ health and environmental welfare, the government should continue to provide television and internet network infrastructures, and provide guidance on internet usage to migrant construction workers.

This paper has two limitations. The first is related to the research design. The employed dataset of this paper used a residential-based sampling method, in which a number of rural migrants may be omitted. As a result, the number of samples included in this study was only 678. Further studies could expand the sample to arrive at more accurate results. In addition, the dataset this paper used is a second-hand data collected by other research institutions. Health and ERP are only part of the questionnaire survey. As such, control variables involved in this paper cannot contain all other factors affecting health, such as workers’ behaviors and safety measures accessibility. The second limitation is related to the method. Considering that pre-existing health or mental health issues may influence ERP, the six dimensions of ERP might be endogenous variables. In this paper, SUR was employed to test the robustness of results. Future research could use panel data which can observe the long-term trends of health and migrant workers’ ERP to solve this problem.

## 5. Conclusions

In conclusion, this paper has shown that migrant construction workers’ perceptions of air pollution, industrial waste pollution, noise pollution, domestic waste pollution, water pollution and food pollution at both their working and living sites, are significantly associated with their physical and mental health. This paper further has shown that the six dimensions of ERP exert heterogeneous influences on the physical and mental health of migrant construction workers in different gender and age groups. In addition to focusing on migrant construction workers’ ERP at working sites, this paper is the first to also assess migrant construction workers’ ERP at their living sites. The results are clear reminders that paying attention to the ERP of migrant construction workers at all their sites of activity (including work and living sites) in the city is necessary and conducive to promoting the physical and mental health of this particular group. This paper contributed to the literature on the influence of ERP on migrant construction workers’ physical and mental health in China.

## Figures and Tables

**Table 1:** Descriptive analysis (*N* = 678).

Variable	Mean	Median	IQR (25–75%)	SD	Min	Max
Physical health	4.10	4	4–5	0.87	1	5
Mental health	4.11	4	4–5	0.88	1	5
Air pollution perception	2.81	3	2–4	1.55	0	5
Industrial waste pollution perception	2.55	3	1–4	1.57	0	5
Noise pollution perception	2.78	3	2–4	1.50	0	5
Domestic waste pollution perception	2.74	3	2–4	1.44	0	5
Water pollution perception	2.86	3	2– 4	1.50	0	5
Food pollution perception	2.81	3	2–4	1.47	0	5
Age (year)	41.02	41	33–49	10.83	17	65
Education (year)	9.27	9	7.5–12	3.37	0	15
Logarithm of income	10.21	10.31	9.9–10.6	0.74	7.6	12.9
Working year (year)	15.24	13	7–21	10.54	1	50
Environmental literacy	4.82	5	3–7	2.37	0	9
Television	4.13	4	4–5	0.90	1	5
Internet	2.68	3	1–4	1.60	1	5
Phone	1.93	1	1–3	1.22	1	5
**Variable**				**Item**	**Freq.**	**Percent**
Gender				Male (“1”)	575	84.81
Ethnicity				Han ethnicity (“1”)	628	92.63

Notes: IQR represents the interquartile range.

**Table 2:** Influences of ERP on migrant construction workers’ physical and mental health (*N* = 678).

Variable	Physical Health	Mental Health
	b Coefficient	b Coefficient
Air pollution perception	0.060 ** (0.028)	0.083 *** (0.027)
Industrial waste pollution perception	0.061 ** (0.028)	0.104 *** (0.027)
Noise pollution perception	0.065 ** (0.029)	0.200 *** (0.029)
Domestic waste pollution perception	0.066 ** (0.030)	0.081 *** (0.029)
Water pollution perception	0.055 * (0.029)	0.066 ** (0.028)
Food pollution perception	0.065 ** (0.030)	0.131 *** (0.029)
Gender (male)	−0.226 * (0.121)	0.013 (0.117)
Age	−0.015 ** (0.006)	0.010 * (0.006)
Ethnic (Han ethnicity)	−0.028 (0.162)	−0.138 (0.161)
Education	−0.002 (0.015)	0.004 (0.014)
Logarithm of income	0.221 *** (0.063)	0.063 (0.061)
Working year	−0.002 (0.005)	−0.005 (0.005)
Environmental literacy	0.011 (0.020)	−0.004 (0.019)
Television	0.139 *** (0.048)	0.063 (0.047)
Internet	−0.001 (0.037)	0.066 * (0.036)
Phone	−0.012 (0.039)	−0.028 (0.037)
Region	Controlled	Controlled
Pseudo-R^2^	0.031	0.015

Notes: Standard errors in brackets; *** *p* < 0.01, ** *p* < 0.05, * *p* < 0.1.

**Table 3:** Influences of ERP on migrant construction workers’ physical and mental health for different gender groups.

Variable	Physical Health		Mental Health	
	Female	Male	Female	Male
	b Coefficient	b Coefficient	b Coefficient	b Coefficient
Air pollution perception	0.142 * (0.084)	0.053 * (0.030)	−0.034 (0.080)	0.096 *** (0.029)
Industrial waste pollution perception	0.157 * (0.082)	0.048 (0.030)	0.033 (0.078)	0.112 *** (0.029)
Noise pollution perception	0.057 * (0.031)	0.103 (0.080)	0.125 (0.079)	0.211 *** (0.031)
Domestic waste pollution perception	0.253 *** (0.084)	0.043 (0.032)	0.217 *** (0.081)	0.068 ** (0.032)
Water pollution perception	0.098 (0.082)	0.053 * (0.031)	−0.054 (0.079)	0.081 *** (0.031)
Food pollution perception	0.055 (0.087)	0.061 * (0.032)	0.090 (0.086)	0.133 *** (0.031)
Age	0.014 (0.018)	−0.021 *** (0.006)	0.012 (0.019)	−0.023 *** (0.006)
Ethnic (Han ethnicity)	−0.459 (0.487)	−0.034 (0.163)	−0.413 (0.516)	0.037 (0.174)
Education	−0.011 (0.037)	−0.003 (0.015)	−0.023 (0.039)	0.001 (0.017)
Logarithm of income	0.029 (0.017)	0.237 *** (0.068)	−0.014 (0.188)	0.223 *** (0.068)
Working year	0.035 * (0.017)	−0.002 (0.005)	0.034* (0.018)	−0.005 (0.006)
Environmental literacy	−0.062 (0.062)	0.012 (0.020)	−0.062 (0.062)	0.016 (0.021)
Television	0.191 (0.156)	0.152 *** (0.052)	0.197 (0.159)	0.154 *** (0.052)
Internet	0.241 ** (0.107)	−0.008 (0.038)	0.222 ** (0.112)	−0.037 (0.041)
Phone	0.122 (0.104)	−0.006 (0.039)	0.119 (0.105)	−0.017 (0.042)
Region	Controlled	Controlled	Controlled	Controlled
*N*	103	575	103	575
Pseudo-R^2^	0.079	0.037	0.060	0.004

Notes: Standard errors in brackets; *** *p* < 0.01, ** *p* < 0.05, * *p* < 0.1.

**Table 4:** Influences of ERP on migrant construction workers’ physical and mental health for different age groups.

Variable	Physical Health		Mental Health	
	Age ≥ 50	Age < 50	Age ≥ 50	Age < 50
	b Coefficient	b Coefficient	b Coefficient	b Coefficient
Air pollution perception	0.116 * (0.064)	0.006 (0.042)	0.044 (0.084)	0.006 (0.042)
Industrial waste pollution perception	0.096 (0.068)	−0.006 (0.042)	−0.035 (0.084)	−0.006 (0.042)
Noise pollution perception	0.182 *** (0.067)	0.204 *** (0.038)	0.157 * (0.082)	0.204 *** (0.038)
Domestic waste pollution perception	0.220 *** (0.073)	0.016 (0.038)	0.063 (0.086)	0.016 (0.038)
Water pollution perception	0.217 *** (0.065)	−0.06 (0.043)	−0.037 (0.091)	−0.060 (0.0430)
Food pollution perception	0.062 (0.069)	0.103 *** (0.036)	0.017 (0.076)	0.103 *** (0.036)
Gender (male)	−0.136 (0.135)	0.084 (0.129)	−0.257 (0.333)	0.084 (0.129)
Age	−0.009 (0.009)	0.011 (0.009)	−0.016 (0.027)	0.011 (0.009)
Ethnic (Han ethnicity)	0.245 (0.181)	−0.106 (0.182)	−0.081 (0.451)	−0.106 (0.182)
Education	0.003 (0.019)	0.008 (0.018)	−0.028 (0.030)	0.008 (0.018)
Logarithm of income	0.116 (0.075)	−0.055 (0.073)	0.266 ** (0.134)	−0.055 (0.073)
Working year	0.002 (0.008)	−0.007 (0.008)	0.004 (0.008)	−0.007 (0.008)
Environmental literacy	0.012 (0.024)	−0.002 (0.023)	−0.030 (0.040)	−0.002 (0.023)
Television	0.149 *** (0.053)	0.064 (0.052)	0.088 (0.146)	0.064 (0.052)
Internet	0.029 (0.043)	0.058 (0.041)	−0.058 (0.113)	0.058 (0.041)
Phone	0.020 (0.042)	0.006 (0.041)	−0.001 (0.129)	0.006 (0.041)
Region	Controlled	Controlled	Controlled	Controlled
*N*	153	525	153	525
Pseudo-R^2^	0.022	0.040	0.027	0.040

Notes: Standard errors in brackets; *** *p* < 0.01, ** *p* < 0.05, * *p* < 0.1.

**Table 5:** Influences of ERP on migrant construction workers’ physical and mental health by SUR (*N* = 678).

Variable	Physical Health	Mental Health
	b Coefficient	b Coefficient
Air pollution perception	0.170 ** (0.069)	0.171 *** (0.050)
Industrial waste pollution perception	0.179 ** (0.069)	0.224 *** (0.048)
Noise pollution perception	0.170 ** (0.066)	0.378 *** (0.047)
Domestic waste pollution perception	0.162 ** (0.064)	0.147 *** (0.048)
Water pollution perception	0.152 * (0.066)	0.121 ** (0.049)
Food pollution perception	0.146 ** (0.065)	0.131 *** (0.029)
Control variables	Controlled	Controlled
R^2^	0.087	0.116
*p* value	0.000	0.000
Breusch-Pagan test	Chi^2^ = 8.372 ***	

Notes: Standard errors in brackets; *** *p* < 0.01, ** *p* < 0.05, * *p* < 0.1.

## Appendix A

**Questions on environmental literacy**

(Answer choices: True/False/Not clear)

1. Automobile exhaust will not pose a threat to human health.
2. Excessive use of chemical fertilizers and pesticides will cause environmental damage.
3. The use of washing powder containing phosphate will not cause water pollution.
4. The fluorine emission of fluorine-containing refrigerators will become a factor to destroy the atmospheric ozone layer.
5. Acid rain has no correlation with coal burning.
6. Species are interdependent, and the disappearance of a species can produce a chain reaction.
7. In the air quality report, grade III air quality is better than grade I air quality.
8. Single species of trees are more likely to cause insect pests.
9. In the water pollution report, the water quality of class V is better than that of class I.
10. The increase of carbon dioxide in the atmosphere will be a factor of climate warming.
